# Self-assessed performance-based function test versus patient-reported outcome measures for knee and hip osteoarthritis

**DOI:** 10.1186/s13102-024-01020-2

**Published:** 2024-11-21

**Authors:** Ali Kiadaliri, Paulina Sirard, Leif E. Dahlberg, L. Stefan Lohmander

**Affiliations:** 1 Department of Clinical Sciences Lund, Orthopaedics, Lund University, Lund, Sweden; 2 Joint Academy®, Malmö, Sweden; 3 Skåne University Hospital, Remissgatan 4, Lund, SE-221 85 Sweden

**Keywords:** 30-second chair stand test, Physical function, HOOS-12, KOOS-12, Digital health, Osteoarthritis

## Abstract

**Background:**

Physical function constitutes a key component of outcome assessment for almost all osteoarthritis interventions. The aim was to compare physical function measured using a self-assessed performance-based test versus self-reported function using questionnaires among individuals with knee or hip osteoarthritis (OA) participating in a digital exercise and education therapy.

**Methods:**

We analysed data from individuals aged 40 + years participating in the digital program. We extracted data on the self-assessed 30-second chair stand test (30s CST) and the function subscales of Knee injury/Hip disability and Osteoarthritis Outcome Score 12 (KOOS-12/HOOS-12) at enrolment and 3- (*n* = 10884) and 12-month (*n* = 3554) follow-ups. Participants completed Numeric Rating Scale (NRS) pain, EQ-5D-5L, and an external anchor: global rating of change scale. Correlations were assessed using the Spearman correlation coefficient, responsiveness using standardized response mean (SRM) and receiver operating characteristic (ROC) curves, and agreement using weighted percent of agreement and weighted Gwet’s agreement coefficient.

**Results:**

Correlations were weak between the 30s CST and KOOS-12/HOOS-12 function (*r* < 0.35 for raw and *r* < 0.20 for change scores). Correlations with NRS pain and EQ-5D-5L were stronger for the KOOS-12/HOOS-12 function subscale than for 30s CST. Greater internal (SRM > 1 vs. SRM < 0.5) and lower external responsiveness were observed for the 30s CST versus the KOOS-12/HOOS-12 function, even though external responsiveness was generally inadequate for both (the area under the ROC curves < 0.7). The direction of change was similar for the two function measures for about 70% of subjects with moderate agreement between them (weighted Gwet’s agreement coefficient range 0.45 to 0.50).

**Conclusion:**

Weak correlations and moderate agreements between function measured using performance-based test and self-reported using KOOS-12/HOOS-12 in people with knee or hip OA suggest that they may capture different aspects of functional abilities in this population.

**Supplementary Information:**

The online version contains supplementary material available at 10.1186/s13102-024-01020-2.

## Background

Osteoarthritis (OA) is a leading cause of pain, disability, and physical function impairment in affected persons [1, 2]. Pain and physical function limitations are associated with increased risk of disability, poor quality of life, cognitive impairment, reduced work productivity and elevated healthcare use [1, 3]. Hence, physical function constitutes a key component of outcome assessment for almost all OA interventions [4] and is recommended by the Osteoarthritis Research Society International (OARSI) and the Outcome Measures in Rheumatology (OMERACT) [5, 6]. Physical function can be measured subjectively using patient-reported outcome measures (PROMs) and objectively using performance-based tests [7]. While PROMs evaluate people‘s own perception of their functional abilities, performance-based tests quantify people’s actual functional performance [8]. In this line, the OARSI recommended a core set of performance-based tests for use in people with OA [6]. While reliability of the OARSI performance-based tests is agreed upon, the findings on their validity and responsiveness are mixed [8, 9]. Previous studies comparing PROMs with performance-based tests in people with OA, generally reported weak to moderate correlations between them [8, 10, 11]. However, most previous studies were conducted in individuals with OA who were waiting for or underwent joint replacement with few studies on persons using non-surgical treatments [10]. Since people on surgical treatment generally have more pain and physical limitations than those on non-surgical treatments, the results from the former cohorts might not be generalizable to the latter cohorts. More importantly, the increase in the use of digitally delivered exercise and education treatments for OA management implies that there will more reliance on remotely self-assessed performance-based tests [12]. To our knowledge, no previous study assessed the relationships between PROMs and self-assessed performance-based measures of physical function and compared their responsiveness among OA patients who are treated digitally. This study aimed to address this knowledge gap by evaluating the correlations, responsiveness and agreement between function measured using the Knee injury and Osteoarthritis Outcome Score 12 (KOOS-12) or Hip disability and Osteoarthritis Outcome Score (HOOS-12) [13] function subscale and patient self-assessment using the 30-second chair-stand test (30s CST) as a performance-based measure [6] in a large cohort of individuals participating in a digitally delivered education and exercise program for knee and hip OA and followed up to one year in Sweden.

## Methods

### Study design and setting

This is a secondary analysis of register data obtained from participants of a digitally delivered education and exercise treatment for hip and knee OA in Sweden, known as Joint Academy^®^, described in detail [14, 15]. In short, participants joined the digital program by recommendation from their physiotherapist, orthopaedic surgeon, or joined through online advertisements and campaigns on search engines and social networks. Participants in the digital program had to have a prior radiographic and/or clinical diagnosis of hip or knee OA from a physiotherapist or physician. Those without a prior diagnosis had their clinical OA confirmed through a telephone consultation or a physical visit with an orthopaedic surgeon or physiotherapist. The program is delivered in the Swedish language and hence a proficient understanding of the Swedish language was required. In addition, having a Swedish social security number and owning a smartphone were additional requirements for participation. The digital program is delivered as a smartphone application based on individualized exercises adjusted with participants’ progression in the program. It also covers lessons on OA, physical activity, and self-management, followed by short quizzes on the topics. Participants receive regular supervision from their physiotherapist via telephone or video calls, with the added option of asynchronous chat communications available throughout the entire duration of the program. While the program can continue upon the participants’ willingness to keep participating, the core content and basic package are delivered within 12 weeks.

### Participants

We extracted data on all consecutive participants of the digital program aged 40 + years who enrolled in the program between January 1st, 2020, and September 30th, 2021 (*n* = 15,944). Of these, we excluded 607 individuals with the missing response to 30s CST and/or KOOS-12/HOOS-12 at enrolment. Additionally, we excluded 3,728 participants with no responses to 30s CST and/or KOOS-12/HOOS-12 at 3- and 12-month follow ups. Data were extracted on October 24th, 2022.

### PROMs-based function

We used KOOS-12 and HOOS-12 Function subscales to measure function among people with knee and hip OA, respectively [12]. The KOOS-12 and HOOS-12 include 12 items of the full KOOS (42 items) and HOOS (40 items) questionnaires. The Function subscale of both KOOS-12 and HOOS-12 covers 4 items from the Activities of Daily Living (ADL) and Sport/Recreation subscales of the original KOOS/HOOS. There are 3 common items in the KOOS-12/HOOS-12 including “rising from sitting”, “standing”, and “getting in/out of car”. The fourth items in the KOOS-12 and HOOS-12 are “twisting/pivoting” and “walk on uneven surface”, respectively. Each item is scored from 0 to 4 and the mean of the function subscale normalized to a score from zero (extreme functional problems) to 100 (no functional problems) [13]. The Swedish versions of KOOS-12/HOOS-12 were used and were responded to digitally by the user through the app.

### Performance-based function

This was measured using the 30s CST, a performance-based test assessing the activity “sit-to-stand movement” [6]. The 30s CST is among the physical function measures recommended by OARSI [6] and has been identified as one of the best rated sit-to-stand tests among people with knee OA [16]. The test is implemented by scoring the maximum number of sit to stand from a chair for 30 s (a full sit-to-stand and consecutive stand-to-sit is counted as one chair stand). In the current study, the participants in the digital program had access to an instruction video and were asked to execute the test using a provided digital stopwatch. The maximum number of repetitions were *self-reported* into the digital platform by the participants. Previous studies reported good inter-rater reliability between self-assessed and physiotherapist-assessed 30s CST [17, 18], albeit only one of these studies [18] explored the reliability of 30s CST administered digitally as a self-test. Notably, the study by Karlsson [18] used the same digital platform as the present study and reported an ICC of 0.930 for inter-rater reliability and an ICC of 0.924 for test-retest reliability, both suggesting the good reliability of the digital assessment of 30s CST as a self-test.

### Other measures

Participants rated their level of knee/hip pain over the previous week using an 11-point numeric rating scale (NRS) for pain (0 = no pain and 10 = the worst possible pain) which is a valid and reliable measure for assessing pain in OA [19]. Health-related quality of life was assessed using the five-level version of EQ-5D (EQ-5D-5L) [20] which has been suggested as a reliable, valid and responsive measure in people with OA [21]. We used the Swedish value set to compute the EQ-5D-5L index score [22]. These were collected at the enrolment and 3- and 12-month follow ups.

Participants responded to an external anchor at follow-ups: global rating of change (GRoC). The GroC asked the participants to rate their physical function at each follow up compared to the enrolment (“How is your ability to perform daily activities now, compared with prior to your participation in the treatment?”), with 7 possible options (“An important improvement”, “Somewhat better, but enough to be an important improvement”, “Very small change, not enough to be an important improvement”, “About the same”, “Very small change, not enough to be an important deterioration”, “Somewhat worse, but enough to be an important deterioration” and “Worse, an important deterioration”) [23]. All the measures were reported digitally by the participants.

### Data analysis

We computed the Spearman correlation coefficient with 95% confidence interval (CI) to determine the correlations between 30s CST and KOOS-12/HOOS-12 with one another and with NRS pain and the EQ-5D-5L index score. The correlation between NRS pain and the EQ-5D-5L with the KOOS-12/HOOS-12 Function subscale and 30s CST was computed to assess how knee pain and quality of life were associated with perceived and performance-based functional abilities. The correlation coefficients were computed for both raw scores at enrolment and follow ups as well as change scores from enrolment. Correlation strength was defined as follows: negligible = 0.00 to 0.19, weak = 0.20 to 0.39, moderate = 0.40 to 0.59, strong = 0.60 to 0.79, and very strong = 0.80 to 1.00 [24].

We assessed *internal responsiveness*, that is the ability to detect change over time, using standardized response mean (SRM) computed as mean change score divided by standard deviation of change score [25]. The value of SRM was interpreted as trivial (< 0·2), small (≥ 0·2 and < 0·5), moderate (≥ 0·5 and < 0·8) and large (≥ 0·8) [25]. We obtained 95% CIs using bootstrapping with 1000 replications. *External responsiveness*, that is the relationship between change in a measure and change in an external criterion, was evaluated using receiver operating characteristic (ROC) curves with GroC as the external criterion. For GroC, we classified those who responded “An important improvement” or “Somewhat better, but enough to be an important improvement” as importantly improved. The areas under the ROC curves (AUC) were calculated to quantify the probability of the change scores in the 30s CST/KOOS-12/HOOS-12 to correctly classify patients according to GroC (importantly improved vs. not importantly improved). We considered an AUC value ≥ 0.7 as adequate external responsiveness [26].

To assess the agreement between the two measures of physical function in terms of the direction of change, we divided the participants into three groups based on their change scores: (1) improved: change > 0, (2) stable: change = 0, and (3) worsened: change < 0. We then calculated weighted percent of agreement and weighted Gwet’s agreement coefficient (Gwet’s AC2) with ordinal weight [27] using “kappaetc” command in Stata [28]. The Gwet’s AC2 value < 0 was interpreted as poor, 0.00–0.20 as slight, 0.21–0.40 as fair, 0.41–0.60 as moderate, 0.61–0.80 as substantial, and 0.81–1.00 as almost perfect [29]. We conducted separate analyses for knee and hip OA as well as 3- and 12-month follow ups. All statistical analyses were implemented in Stata v.18.

## Results

After excluding 4,335 individuals with missing responses at enrolment and/or follow ups, a total of 11,609 individuals were included. The characteristics of included and excluded individuals were comparable (i.e. standardized mean difference < 0.1) with the former being slightly older (Table A1 in supplement). Among those included, 8,055 individuals provided the responses only at the 3-month follow up, 725 only at 12-months and 2,829 at both follow ups. Therefore, 10,884 individuals with mean (SD) age 64.8 (8.7), 75.2% females and 59.3% with knee OA were included at the 3-month follow up and 3,554 with mean (SD) age 65.5 (8.4), 73.9% females and 61.1% with knee OA at the 12-month follow up (Table 1). Adherence to the treatment, defined as the weekly average of the percentage of completed activities (exercises, text or video lessons, and quizzes on lesson material) over 12 and 48 weeks of participation in the program, were around 89% in both knee and hip OA cohorts.

**Table 1:** The baseline characteristics of sample included at 3- and 12-month follow up analyses

	Knee osteoarthritis		Hip osteoarthritis		Total	
	3-month	12-month	3-month	12-month	3-month	12-month
N	6450	2173	4434	1381	10,884	3554
Age, mean (SD)	64.8 (8.6)	65.6 (8.4)	64.7 (8.9)	65.3 (8.5)	64.8 (8.7)	65.5 (8.4)
Female, n (%)	4746 (73.6)	1574 (72.4)	3442 (77.6)	1051 (76.1)	8188 (75.2)	2625 (73.9)
Body mass index, mean (SD)	27.5 (4.8)	27.2 (4.6)	26.4 (4.3)	26.3 (4.2)	27.0 (4.6)	26.8 (4.5)
Education, n (%)						
Less than high school	592 (9.2)	208 (9.6)	424 (9.6)	149 (10.8)	1016 (9.3)	357 (10.1)
High school	2361 (36.6)	771 (35.5)	1688 (38.1)	490 (35.5)	4049 (37.2)	1261 (35.5)
College/university	3497 (54.2)	1194 (55.0)	2322 (52.4)	742 (53.7)	5819 (53.5)	1936 (54.5)
30-second chair stand test, mean (SD)	12.8 (4.3)	12.7 (4.3)	13.1 (4.3)	12.9 (4.4)	12.9 (4.3)	12.8 (4.4)
KOOS-12 function scale (0–100), mean (SD)	61.7 (19.2)	62.1 (19.2)	NA	NA	61.7 (19.2)	62.1 (19.2)
HOOS-12 function scale (0–100), mean (SD)	NA	NA	63.4 (18.8)	64.4 (18.4)	63.4 (18.8)	64.4 (18.4)
Pain (NRS, 0–10), mean (SD)	5.1 (1.9)	5.1 (1.9)	5.1 (1.9)	5.0 (1.9)	5.1 (1.9)	5.0 (1.9)
EQ-5D-5L index score (-0.31–1), mean (SD)	0.85 (0.16)	0.85 (0.16)	0.84 (0.17)	0.86 (0.16)	0.85 (0.17)	0.85 (0.16)

SD: standard deviation; KOOS-12: Knee injury and Osteoarthritis Outcome Score-12; HOOS-12: Hip disability and Osteoarthritis Outcome Score-12; NRS: Numeric rating scale

There were weak correlations between 30s CST and KOOS-12/HOOS-12 function subscale raw scores at both follow ups, while the correlations between their change scores were negligible (Table 2). As expected, KOOS-12/HOOS-12 function had stronger correlations with NRS pain and the EQ-5D-5L index score than the 30s CST. The results were similar for knee and hip OA.

**Table 2:** Spearman rank correlation coefficients (95% confidence intervals) between measures of function and other patient-reported outcome measures

	Knee osteoarthritis		Hip osteoarthritis			
	3-month	12-month	3-month		12-month	
30s CST vs. KOOS-12/HOOS-12 function, raw scores ^a^	0.28 (0.26, 0.31)	0.32 (0.28, 0.36)	0.29 (0.26, 0.32)		0.31 (0.26, 0.36)	
30s CST vs. KOOS-12/HOOS-12 function, change scores ^a^	0.13 (0.10, 0.15)	0.16 (0.12, 0.20)	0.13 (0.10, 0.16)		0.15 (0.10, 0.20)	
30s CST vs. NRS pain, raw scores	-0.24 (-0.26, -0.21)	-0.29 (-0.33, -0.25)	-0.25 (-0.28, -0.22)		-0.27 (-0.32, -0.22)	
KOOS-12/HOOS-12 function vs. NRS pain, raw scores ^a^	-0.65 (-0.66, -0.63)	-0.63 (-0.66, -0.61)	-0.66 (-0.68, -0.64)		-0.67 (-0.70, -0.64)	
30s CST vs. NRS pain, change scores	-0.18 (-0.21, -0.16)	-0.25 (-0.29, -0.21)	-0.18 (-0.21, -0.15)		-0.22 (-0.27, -0.17)	
KOOS-12/HOOS-12 function vs. NRS pain, change scores ^a^	-0.46 (-0.48, -0.44)	-0.49 (-0.52, -0.46)	-0.44 (-0.46, -0.42)		-0.50 (-0.54, -0.46)	
30s CST vs. EQ-5D-5L, raw scores	0.24 (0.21, 0.26)	0.26 (0.23, 0.30)	0.26 (0.24, 0.29)		0.29 (0.24, 0.33)	
KOOS-12/HOOS-12 function vs. EQ-5D-5L, raw scores ^a^	0.65 (0.63, 0.66)	0.66 (0.64, 0.69)	0.68 (0.67, 0.70)		0.69 (0.66, 0.71)	
30s CST vs. EQ-5D-5L, change scores	0.10 (0.08, 0.12)	0.19 (0.15, 0.23)	0.11 (0.08, 0.14)		0.13 (0.08, 0.18)	
KOOS-12/HOOS-12 function vs. EQ-5D-5L, change scores ^a^	0.39 (0.37, 0.41)	0.44 (0.41, 0.48)	0.39 (0.36, 0.41)		0.48 (0.44, 0.52)	

30s CST: 30-second chair stand test; KOOS-12: Knee injury and Osteoarthritis Outcome Score-12; HOOS-12: Hip disability and Osteoarthritis Outcome Score-12; NRS: Numeric rating scale

^a^ KOOS-12 (HOOS-12) was used among people with knee (hip) osteoarthritis

The number of 30s CST rose, on average, by 5–7 repetitions between enrolment and follow ups (Table 3). The mean changes in the KOOS-12 and HOOS-12 function scales were about 7–8 points at follow ups. The SRM was large (≥ 0·8) for the performance-based test and small (≥ 0·2 and < 0·5) for the KOOS-12 and HOOS-12 function. On the other hand, the KOOS-12 and HOOS-12 function showed greater external responsiveness than the 30s CST, even though both measures had inadequate external responsiveness (AUC < 0.7).

**Table 3:** The internal and external responsiveness of self-assessed performance-based test and patient-reported outcome measure and agreement between them

	3-month		12-month	
	30s CST	KOOS-12/HOOS-12 function ^a^	30s CST	KOOS-12/HOOS-12 function ^a^
**Knee osteoarthritis**				
Mean change (SD)	5.2 (4.6)	7.4 (16.4)	6.9 (5.3)	7.9 (18.0)
Standardized response mean	1.13 (1.09, 1.17)	0.45 (0.43, 0.48)	1.30 (1.23, 1.37)	0.44 (0.40, 0.48)
AUC for global rating of change	0.58 (0.56, 0.60)	0.67 (0.65, 0.68)	0.65 (0.62, 0.68)	0.70 (0.68, 0.73)
Weighted percent of agreement	69.0 (67.9, 70.0)%		69.5 (67.7, 71.2)%	
Weighted Gwet’s Agreement Coefficient	0.48 (0.46, 0.50)		0.51 (0.47, 0.54)	
**Hip osteoarthritis**				
Mean change (SD)	5.2 (4.8)	6.7 (15.7)	7.1 (5.4)	6.5 (17.6)
Standardized response mean	1.08 (1.04, 1.12)	0.43 (0.40, 0.46)	1.31 (1.24, 1.39)	0.37 (0.31, 0.42)
AUC for global rating of change	0.60 (0.58, 0.62)	0.66 (0.65, 0.68)	0.59 (0.56, 0.63)	0.71 (0.68, 0.74)
Weighted percent of agreement	68.0 (66.7, 69.2)%		66.0 (63.7, 68.2)%	
Weighted Gwet’s Agreement Coefficient	0.46 (0.43, 0.48)		0.44 (0.39, 0.48)	

SD: standard deviation; AUC: Area under the curve; 30s CST: 30-second chair stand test; KOOS-12: Knee injury and Osteoarthritis Outcome Score-12; HOOS-12: Hip disability and Osteoarthritis Outcome Score-12

Values in parentheses display 95% confidence intervals unless otherwise stated

^a^ KOOS-12 (HOOS-12) function was used among people with knee (hip) osteoarthritis

Among persons with knee (hip) OA, 88.5% (87.5%) and 91.3% (92.0%) reported positive changes (> 0) in 30s CST at 3- and 12-month follow ups, respectively (Tables A2&A3 in supplement). The corresponding figures for the KOOS-12 (HOOS-12) function subscale were 57.9% (57.3%) at 3-month and 58.5% (55.2%) at 12-month follow ups. The two measures agreed on the direction of change in about 66–69% of the participants with the weighted Gwet’s AC2 values of 0.44 to 0.51 suggesting moderate agreements (Table 3).

## Discussion

We explored the connection between physical function measured using a self-reported performance-based test versus the KOOS-12/HOOS-12 function subscale in people with mild to moderate knee or hip OA severity participating in a digitally delivered education and exercise therapy and compared their internal and external responsiveness. The findings of this observational study documented weak correlations and moderate agreements between these measures in this cohort. This suggests that these measures capture different aspects of functional abilities and supports the use of both measures to capture physical function in this population.

The weak correlations between performance-based and PROMs-based measure of function reported in the present study are consistent with previous studies conducted among persons with OA [10, 11, 30, 31, 32]. Specifically for 30s CST, previous studies reported correlations between 0.04 [9] and 0.62 [33] with WOMAC physical function subscale, 0.20 to 0.40 with KOOS–/HOOS–Physical Function Short Form (KOOS–PS/HOOS–PS) [8, 34, 35], 0.37 with KOOS ADL subscale [10] and 0.51 (95% CI 0.38, 0.61) with KOOS-12 function subscale [35]. Negligible to weak correlations were also reported between change scores of 30s CST and KOOS-12 function, KOOS–PS and HOOS–PS [8,36,37]. These generally weak to moderate correlations between performance-based measures and PROMs-based function suggest that they are capturing different aspects of functional impairment [8, 10]. Combining these weak correlations with the observed moderate agreement between the two measures in the direction of change in the present study highlights the importance of including both measures of physical function in clinical studies assessing the effectiveness of OA treatments.

The stronger correlations observed between the KOOS-12/HOOS-12 function subscale and other patient-reported outcome measures (i.e. NRS pain and EQ-5D-5L) were in line with previous findings [36, 37–39]. This was expected since all these measures (PROMs) were subjective measures capturing how people feel about their symptoms and functions in daily life, while performance-based tests (e.g. 30s CST) capture how well they can perform a task during a single assessment [37]. Moreover, PROMs for measuring function have been suggested to be intrinsically pain-dominated (i.e. participants’ perceptions of their functional abilities are substantially influenced by their pain level) [40, 41]. In contrast, knee pain and quality of life seems to have limited influence on functional performance of the participants.

Mixed findings have been reported regarding the internal responsiveness of performance-based tests versus PROMs-based function. For example, both higher [37, 42, 43] and lower [33, 44] internal responsiveness of PROMs- than performance-based function has been reported. We observed greater external responsiveness for function measured using PROMs than performance-based measure. Previous studies yielded mixed findings on the topic reporting both similar [45, 46] and different [33, 37] external responsiveness of performance-based tests and PROMs-based function among people with knee OA or hip fracture. Moreover, both adequate [45] and inadequate external responsiveness [37, 46] have been documented. Differences in setting, population, interventions, function measures used, and external anchors might partially explain the cross-study differences in the findings. For instance, Gill et al. [33] reported mixed findings on external responsiveness of 30s CST versus different PROMs-based function in the same cohort. Inadequate external responsiveness observed in the present study might reflect the recall bias of the anchor-based methods. This recall bias suggests that patients’ current health status influences their responses more strongly than the amount of change from baseline [47]. Inadequate responsiveness of the 30s CST, and especially the KOOS-12/HOOS-12 function subscale, in the present study, highlights the need for assessing the responsiveness of these measures among people with mild to moderate OA severity participating in first-line OA treatments in future studies. This inadequate responsiveness might also be due to small to moderate effect of education and exercise therapy on pain and physical function as suggested in a recent systematic review [48]. In a head-to-head comparison of education and exercise versus total knee arthroplasty, Young et al. reported 9.7 versus 30.7 units changes in KOOS-12 function at 1-year following treatments [49]. However, the moderate intervention effect is unlikely to fully explain the inadequate responsiveness considering that the NRS pain demonstrated large internal (SRM > 0.9) and adequate external responsiveness (AUC ranging 0.73 to 0.77) on all occasions among the participants of the present study.

To our knowledge, this is the first study exploring the relationships between digital self-assessment of 30s CST and the KOOS-12/HOOS-12 function subscale among persons with knee or hip OA. Large sample size, exploring correlations, responsiveness and agreements of these measures with data up to 1-year follow ups are other main strengths of the present study. Several limitations of the current study should be acknowledged. The data on 30s CST was measured in the participant’s home and was self-reported which might differ from those measured by healthcare professionals in a clinical setting, albeit two recent studies reported good agreement between self-reported and physiotherapist-measured 30s CST [17, 18], even though this doesn’t rule out the possibility of the difference in the present study. This implies that our results may not be generalizable to the 30s CST assessed by healthcare professionals. The use of GRoC to categorize participants into “importantly improved” and “not importantly improved” groups is prone to recall bias [50]. The majority (74%) of the participants reported a mild to moderate level of NRS pain (i.e. <7 [51]) at baseline which limits the generalizability of the findings for those with more severe OA pain. The non-random nature of voluntary self-selection into the digital treatment means that the participants are different from those identified in routine clinical practice, with more females and high educated among those participating in the digital program, which could limit the generalizability of the findings [52].

## Conclusion

We observed weak correlations and moderate agreements between a self-assessed 30s CST and the KOOS-12/HOOS-12 function subscale to measure physical function in people with mild to moderate knee or hip OA severity participating in a digital exercise and education treatment. The findings support the use of both performance- and PROM-based measures in clinical settings for a more comprehensive assessment of physical function in individuals with mild to moderate knee or hip OA and to achieve a better understanding of OA treatment progress. Inadequate responsiveness highlights the need for developing and validating alternative measures of physical function for digital education and exercise interventions. Specifically, further studies should explore internal and external responsiveness of the KOOS-12/HOOS-12 function subscales among people with less severe knee or hip OA undergoing non-surgical OA treatments.

## Electronic supplementary material

Below is the link to the electronic supplementary material.

Supplementary Material 1

## Data Availability

The data that support the findings of this study are available from Joint Academy^®^ but restrictions apply to the availability of these data, which were used under ethical permission for the current study, and so are not publicly available. Data may be made available through the corresponding author upon reasonable request and with permission of Joint Academy^®^.

## Abbreviations

30s CST: 30-second chair stand test
HOOS-12: Hip disability and Osteoarthritis Outcome Score-12
KOOS-12: Knee injury and Osteoarthritis Outcome Score-12
NRS: Numeric rating scale
OA: Osteoarthritis
PROMs: Patient-reported outcome measures
