# Supplementary material for: Self-assessed performance-based function test versus patient-reported outcome measures for knee and hip osteoarthritis
Source: BMC Sports Sci Med Rehabil. 2024 Nov 21;16:232. doi: 10.1186/s13102-024-01020-2 (PMC11580485; doi:10.1186/s13102-024-01020-2)
Supplement: Supplementary file 1 — Supplementary Material 1 [file 13102_2024_1020_MOESM1_ESM.pdf]

Table A1. The baseline characteristics of excluded and included participants.

	Excluded	Included	Absolute standardized difference
N	4,335	11,609	-
Age, mean (SD)	63.7 (9.5)	64.7 (8.7)	0.11
Female, n (%)	3,226 (74.4)	8,707 (75.0)	0.01
Body mass index, mean (SD)	27.4 (5.0)	27.0 (4.6)	0.07
Education, n (%)			0.08
Less than high school	350 (8.1)	1,074 (9.3)	
High school	1,478 (34.1)	4,301 (37.1)	
College/university	2,463 (56.8)	6,234 (53.7)	
Missing	44 (1.0)	0 (0.0)	
Index joint, n (%)			0.03
Knee	2,511 (57.9)	6,917 (59.6)	
Hip	1,824 (42.1)	4,692 (40.4)	
30-second chair stand test, mean (SD)	12.5 (4.5)[a]	12.9 (4.3)	0.09
KOOS-12 Function scale (0-100), mean (SD)[b]	62.1 (19.3)[c]	61.7 (19.3)[c]	0.02
HOOS-12 Function scale (0-100), mean (SD)[d]	62.9 (19.7)[e]	63.5 (18.8)	0.03
Pain (NRS, 0-10), mean (SD)	5.1 (2.0)[a]	5.1 (1.9)	0.01
General health (NRS, 0-10), mean (SD)	6.5 (1.9)[a]	6.6 (1.8)	0.09

[a] N=4291

[b] Among those with knee osteoarthritis.

[c] N=2135 for excluded and N=6917 for included.

[d] Among those with hip osteoarthritis.

[e] N=1593 for excluded and N=4692 for included.

Table A2. The number of participants with knee osteoarthritis with improved, stable, and worsened function using performance-based test and patient-reported outcome measure.

KOOS-12 / 30s CST	Change <0	Change = 0	Change >0	Total
3-month				
Change <0	149	82	224	455
Change = 0	101	57	128	286
Change >0	1350	976	3383	5709
Total	1600	1115	3735	6450
12-month				
Change <0	51	19	52	122
Change = 0	24	10	33	67
Change >0	481	316	1187	1984
Total	556	345	1272	2173

30s CST: 30-second chair stand test; KOOS-12: Knee injury and Osteoarthritis Outcome Score-12.

Table A3. The number of participants with hip osteoarthritis with improved, stable, and worsened function using performance-based test and patient-reported outcome measure.

HOOS-12 / 30s CST	Change <0	Change = 0	Change >0	Total
3-month				
Change <0	128	53	174	355
Change = 0	67	34	98	199
Change >0	955	658	2267	3880
Total	1150	745	2539	4434
12-month				
Change <0	24	13	39	76
Change = 0	12	5	17	34
Change >0	343	221	707	1271
Total	379	239	763	1381

30s CST: 30-second chair stand test; HOOS-12: Hip disability and Osteoarthritis Outcome Score-12.
